# A unique echinoderm NLR triggers *Vibrio* phagocytosis by promoting microtubule severing to facilitate microfilament polymerization

**DOI:** 10.1016/j.jbc.2026.111418

**Published:** 2026-04-01

**Authors:** Jiaqian Zhu, Kaiyu Chen, Yuxin Li, Yuxuan Liang, Chenghua Li

**Affiliations:** 1 State Key Laboratory of Agricultural Products Safety, Ningbo University, Ningbo, PR China; 2 School of Marine Science and Engineering, Qingdao Agricultural University, Qingdao, PR China; 3 Laboratory for Marine Fisheries Science and Food Production Processes, Qingdao National Laboratory for Marine Science and Technology, Qingdao, PR China

**Keywords:** microtubule-severing proteins, microtubule cytoskeleton, phagocytosis, NOD-like receptor

## Abstract

Mammal nucleotide-binding leucine-rich repeat (NLR) immune receptors detect intracellular pathogen effectors and activate immunity. The origin of mammalian NLRs is thought to be closely related to NLR proteins in metazoans and bacteria, where some novel NLRs encoding NACHT domains combined with other domains have been discovered. However, their functions remain largely unknown. Here we report the first AjNLR-related protein (AjNLR-new) in *Apostichopus japonicus* with an extracellular NACHT domain, which functions as a membrane receptor of *Vibrio splendidus* (AJ01) to regulate phagocytosis. Mechanistically, the regulation of AJ01 phagocytosis by AjNLR-new relies on its intracellular Ig domain recruiting the microtubule-severing protein AjFidgetin, thereby inducing microtubule severing. This leads to microtubule depolymerize around AJ01, forming vacuoles. Surprisingly, at the site of the microtubule vacuole, F-actin polymerize extensively to envelop AJ01, which depends on the direct interaction between the Ig domain of AjNLR-new and actin. Intriguingly, F-actin polymerization during this process is significantly inhibited by interference with AjNLR-new and AjFidgetin or treatment with the microtubule stabilizer (paclitaxel), whereas the microfilament aggregation inhibitor CK666 does not affect microtubule depolymerization. Ultimately, the phagocytosed AJ01 was cleared *via* the lysosomal pathway. We have not only revealed a novel mechanism by which AjNLR-new induces microtubule disassembly through the recruitment of AjFidgetin, thereby promoting F-actin polymerization and facilitating phagocytosis of AJ01, but have also further further enriched and advanced the structural and functional diversity of proteins encoding the NACHT domain.

In the innate immune system, pattern recognition receptors (PRRs) function as sensors for pathogen-associated molecular patterns (PAMPs) that are molecular structures found in a variety of pathogens and damage-associated molecular patterns that are molecules released from damaged cells. Nowadays, several classes of PRRs have been fully characterized, and PRRs can be classified into transmembrane receptor and cytoplasmic receptor based on their location. Transmembrane receptor includes Toll-like receptors (TLRs), Scavenger receptors (SRs), and C-type lectin-like receptors (CLRs) etc (1, 2, 3, 4). Cytoplasmic receptor includs RIG-I-like receptors (RLRs), NOD-like receptors (NLRs) and so on (5, 6, 7). Among them, nucleotide-binding leucine-rich repeat receptors (NLRs), as key sensors of the innate immune system, constitute a large and functionally diverse family of immune receptors in the biological world and are extremely attractive.

In vertebrates, NLR receptors contain three characteristic domains: the NACHT domain enables the activation of the signaling complex *via* ATP-dependent oligomerization, which is essential for the downstream functions of several NLRs (8, 9, 10, 11). The LRR domain at the C-terminus is mainly responsible for recognizing and binding to PAMPs and DAMPs and then mediating the interaction between NLRs and downstream adaptor proteins to activate immune cascades (12, 13). The N-terminal is a protein-protein interaction domain, which mainly mediates downstream signaling through homotypic domains. Based on the difference in N-terminal effector domain, the NLR family in vertebrate is divided into five core subclasses in vertebrates, namely: NLRA with an acidic transactivation domain (AD), NLRB with a baculovirus inhibitory protein repeat domain (BIR), NLRC with a caspase activation and recruitment domain (CARD), NLRP with a pyrin (PYD) domain, and NLRX with an N-terminal unknown domain (14). Activation of some NLRs leads to the formation of multiprotein complexes (called inflammasomes) and the activation of caspase-1, ultimately leading to the production of inflammatory cytokines or the induction of apoptosis or pyroptosis (15). In addition, NLRs also activate NF-κB and MAPK-dependent signaling by interacting with receptor-interacting protein 2 (RIP2) kinase (16). Vertebrate NLRs have been the focus of research, particularly the link between dysfunctional NLRs and a variety of human diseases (17, 18). In contrast, invertebrate NLRs are less well characterized, likely due in part to the lack of NLRs in the classic invertebrate model organisms *Drosophila* and *C*. *elegans* (19). Consequently, research on invertebrate NLRs is still in its early stages.

A large number of NLRs have been found in basal metazoans such as corals, sponges, anemones (20), and hydras. The domain combinations of these invertebrate NLRs are highly dynamic. In addition to the core NACHT domain, a large range of domain loss and recombination events have occurred, resulting in the generation of many NLRs with novel domains and NACHT domain combinations. For example, a unique combination of NACHT domain and glycosyltransferase 1 (Glycos_transf_1) domain appeared in *Acropora digitifera* (21); a combination of NACHT domain and DED domain appeared in Hydra (22); In recent years, numerous novel genes containing the NACHT domain have also been identified in prokaryotic bacteria (23). The role of NLRs with these novel and unique domain combinations in innate immunity remains largely unknown. Interestingly, in our previous work, a novel cell membrane-localized NLR (AjNLRC4) was identified in sea cucumber (*Apostichopus japonicus*) (24, 25), an economical aquaculture species in East Asia. This unique type of NLR is composed of an Ig domain, a NACHT domain, and transmembrane domain (TM domain), which is distinct from the classic cytoplasmic NLRs and through induced cytoskeletal rearrangements by regulating the polymerization of F-actin, thereby regulate the phagocytosis of *Vibrio splendidus*, a major pathogen of sea cucumbers (25). This discovery demonstrates that this sea cucumber NLR with a unique type of domain combination plays an important role in cytoskeletal regulation. However, studies on the role of microtubules in phagocytosis are limited. A major function of microtubules in phagocytosis is vesicle transport, which supports phagosome maturation for target degradation (26). Besides, the role of microtubules play in the initial stage of phagocytosis is unclear. Here, we identified a novel atypical NLR in the sea cucumber NLR repertoire consisting of an Ig domain, a NACHT domain, and TM domain. We named it AjNLR-new, which confirmed it was a *bona fide* membrane PRR with extracellular NACHT domain and intracellular Ig domain. During the infection with *V*. *splendidus*, AjNLR-new acts as a receptor for *V*. *splendidus* and recruits the microtubule-severing protein AjFidgetin to induce microtubule severing, regulating microtubule dynamic instability, thereby mediating the phagocytosis of *V*. *splendidus*. All these results enhance our understanding of the structural innovation and functional diversity of NLRs in invertebrates, as well as our comprehension of the mutual regulatory mechanisms between microtubules and microfilaments during phagocytosis.

## Results

### AjNLR-new is a membrane receptor composed of an extracellular NACHT domain and an intracellular Ig domain

**Figure 1:**
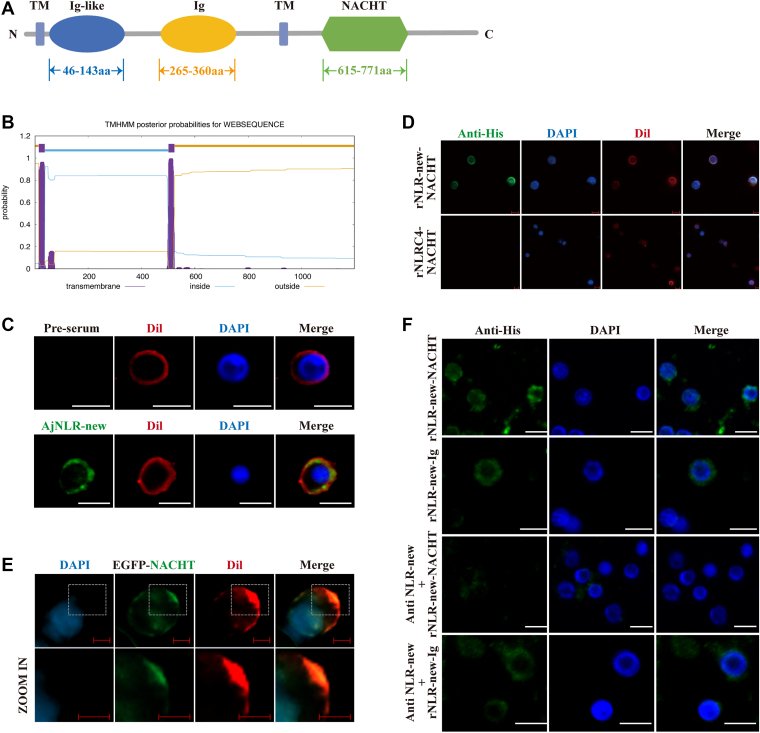
**AjNLR-new is located in the membrane of sea cucumber coelomocytes**. *A*, the domain architecture of sea cucumber AjNLR-new predicted by SMART (http://www.smart.embl-heidelberg.de/). *B*, predicting the transmembrane domain of AjNLR-new using TMHMM (https://services.healthtech.dtu.dk/services/TMHMM-2.0/). *C*, Analysis of AjNLR-new subcellular distribution in coelomocytes. Coelomocytes were subjected to immunofluorescence staining with the cell membrane probe Dil. AjNLR-new is localized to the cell membrane and precisely colocalized with Dil signals. Scale bar = 5 μm. *D*, coelomocyte binding of rAjNLRC4-NACHT and rAjNLR-new-NACHT. rAjNLRC4-NACHT and rAjNLR-new-NACHT were injected into sea cucumbers, and coelomocytes were isolated for immunohistochemistry assays to detect binding abilities with an anti-His tag antibody (*green*). rAjNLR-new-NACHT binds to the coelomocyte surface, and rAjNLRC4-NACHT cannot bind to the coelomocyte surface. Scale bar = 5 μm. *E*, subcellular localization analysis of AjNLR-new-NACHT with TM domain in 293T cells. Scale bar = 10 μm. *F*, differences in the coelomocytes binding ability of AjNLR-new-NACHT and AjNLR-new-Ig after antibody AjNLR-new blocked coelomocytes. rAjNLR-new-Ig and rAjNLR-new-NACHT were injected into sea cucumbers, and coelomocytes were isolated for immunohistochemistry assays to detect binding abilities with an anti-His tag antibody (*green*). Scale bar = 5 μm. The immunoblot data are representative images from one of three independent experiments. n = 3 independent experiments (*C*–*F*) with similar results. Nuclei were stained with DAPI (*blue*).

Unique genes containing a combination of Ig domain, TM domain, and NACHT domain were identified in *A*. *japonicus* (AjNLRC4) (24). We screened these genes using TB Tools and identified a gene with a unique N-terminal Ig domain located within two transmembrane domains and a C-terminal NACHT domain. We named it AjNLR-new (Fig. 1*A*). The open reading frame (ORF) region of AjNLR-new encoded a protein of 1197 amino acids, with a theoretical molecular mass of 136 kDa and an isoelectric point of 5.34 (PIK48001.1) (Fig. S1*A*). The expression patterns of AjNLR-new mRNA in different tissues of *A*. *japonicus* were analyzed by real-time PCR and the Ajβ-actin gene was served as an internal reference. As shown in Figure S1*B*, AjNLR-new was commonly expressed in all detected tissues. After immersion infection with AJ01 in *vivo*, the expression level of AjNLR-new in coelomocytes was significantly increased (Fig. S1*C*). The above results suggest that the upregulation of AjNLR-New was related to AJ01 infection in sea cucumber. We further predict its domain distribution through an online prediction website for transmembrane domains. The results showed that the Ig-like and Ig domains were located inside the cell membrane, while the NACHT domain was located outside the cell membrane (Fig. 1*B*). To confirm the true localization of AjNLR-new in coelomocytes, localization analysis of AjNLR-new using specific antibodies. The results show that green fluorescence signals of AjNLR-new are concentrated in the coelomocyte membrane, which can be colocalized with the membrane-specific indicator Dil (Fig. 1*C*). To determine whether the NACHT domain of AjNLR-new is located extracellularly (unlike AjNLRC4), we investigated the cell-binding abilities of rAjNLR-new-EX (NACHT domain) and rAjNLRC4-IN (NACHT domain). Obvious green signals are observed on the surfaces of coelomocytes injected with recombinant AjNLR-new-EX, but no signals are observed on the surfaces of coelomocytes injected with recombinant rAjNLRC4-IN (Fig. 1*D*). In addition, we transfected the AjNLR-new fragment containing the last TM domain and the NACHT domain into 293T cells to further verify its subcellular localization and found that it also colocalized with Dil (Fig. 1*E*). Finally, we compared the cell binding abilities of rAjNLR-new- EX and rAjNLR-new-IN before and after blocking with rAjNLR-new antibody. Antibody blocking can significantly block the coelomocyte binding ability of rAjNLR-new-EX, while the coelomocyte binding ability of rAjNLR-new-IN, which has very little coelomocyte binding ability, is not affected (Fig. 1*F*).

### The NACHT domain of AjNLR-new performed immune recognition functions and exhibited bacterial agglutination activities in Ca^2+^-dependent manners

**Figure 2:**
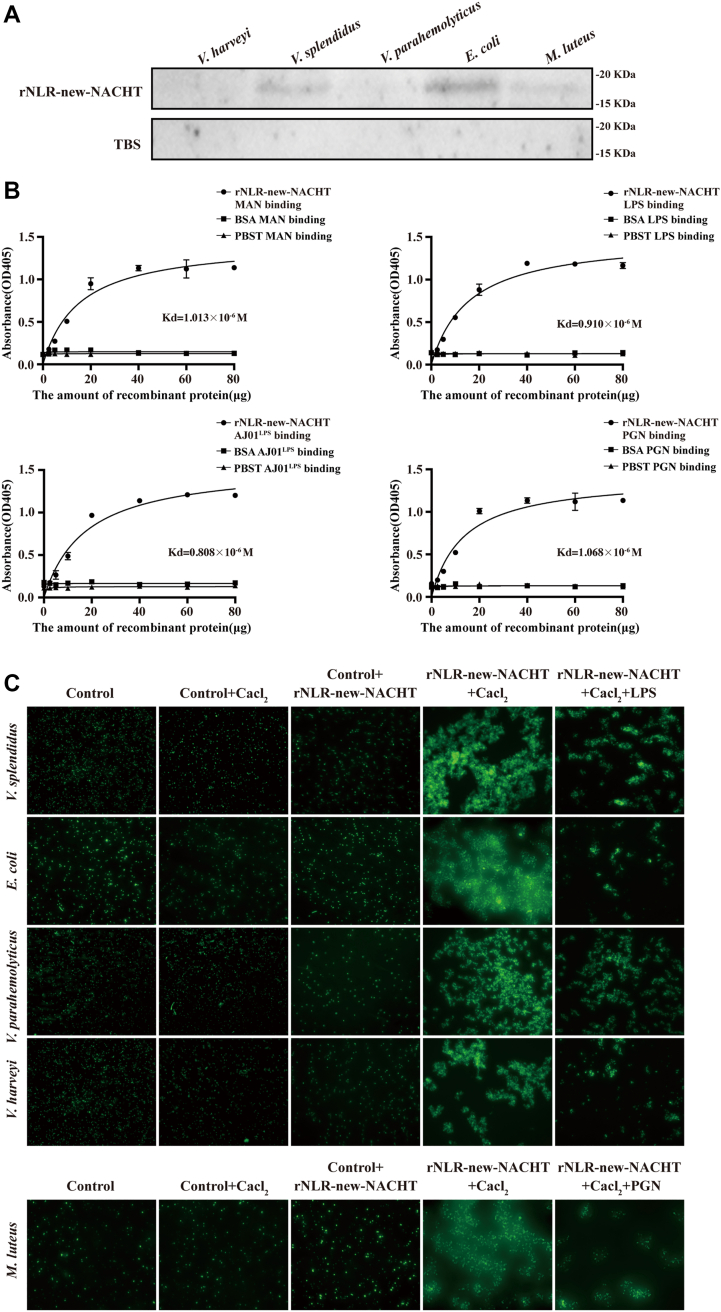
**The NACHT domain of AjNLR-new performs an immune recognition function**. *A*, the bacterial binding activities of rAjNLR-new-NACHT was analyzed by using western blotting. Different bacteria were incubated with rAjNLR-new-NACHT, washed with PBS four times and then analyzed by western blotting with an anti-His antibody. TBS instead of rAjNLR-new-NACHT was used as the negative control. *B*, the binding activities of rAjNLR-new-NACHT to different polysaccharides were analyzed with ELISA. Four polysaccharides were used for ELISA analysis (n = 5), including LPS from *E*. *coli* or AJ01, PGN and MAN. BSA instead of rAjNLR-new-NACHT was used as the negative control. *C*, rAjNLR-new-NACHT agglutinates different microbes in the presence of Ca^2+^. FITC-labeled microbes (*green*) were mixed with an equal volume of rAjNLR-new-NACHT (1 mg/ml) in the presence or absence of 10 mM CaCl_2_ and incubated at room temperature for approximately 1 h. After incubation, agglutination reactions were observed under a fluorescence microscope.

Membrane receptor activation is inseparable from the recognition of pathogens and PAMP by its extracellular domain (27, 28). The NACHT domain has been shown to recognize multiple PAMP (24). Therefore, we analyze the potential abilities of rAjNLR-new-EX bind to bacteria *in vitro*. Western blotting assays reveal that rAjNLR-new-EX could significantly bind to all of the tested microbial strains, including G^+^ bacteria (*Micrococcus luteus*) and G^-^ bacteria (*Vibrio harvey*, *Vibrio parahemolyticus*, *V*. *splendidus* and *Escherichia coli*) (Fig. 2*A*). To elucidate whether the microbial binding ability of rAjNLR-new-EX is mediated by cell surface polysaccharides, ELISAs are performed to detect the capacity of rAjNLR-new-EX to bind to LPS, PGN and MAN. The results indicate that rAjNLR-new-EX could bind to all of these ligands with high affinity (Fig. 2*B*). FITC-labeled microbes are incubated with the tested proteins to check the possible agglutination activity. Immunofluorescence microscopy analysis demonstrates the strong agglutinating activities of rAjNLR-new-EX toward G^+^ bacteria (*M*. *luteus*) and G^-^ bacteria (*V*. *harvey*, *V*. *parahemolyticus*, *V*. *splendidus and E*. *coli*) in the presence of CaCl_2_. Consistently, rAjNLR-new-EX fails to agglutinate all of the tested bacteria in the absence of CaCl_2_. More importantly, rAjNLR-new-EX loses its ability to bind to gram-negative bacteria after the addition of LPS, even in the presence of CaCl_2_ (Fig. 2*C*).

### AjNLR-new acts as a receptor to mediate phagocytosis of AJ01

**Figure 3:**
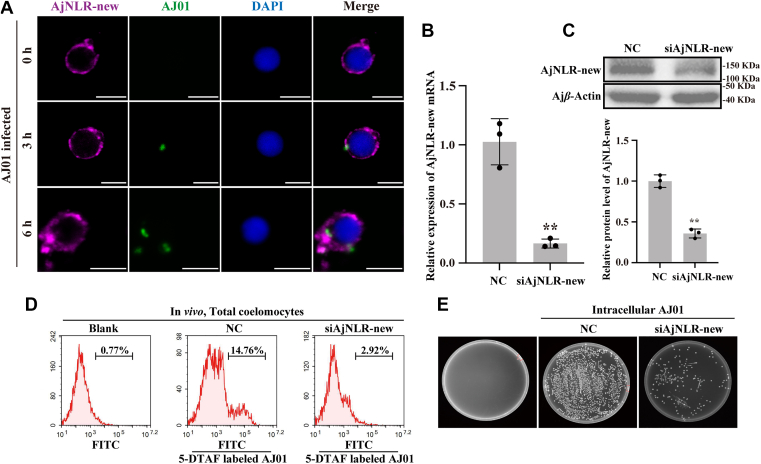
**AjNLR-new acts as a receptor of AJ01 and mediates its phagocytosis**. *A*, immunocytochemistry was used to detect the colocalization of AjNLR-new and 5-DTAF-labeled AJ01 in coelomocytes. The coelomocytes were collected at different time points (0, 3, and 6 h) post-AJ01 injection. Scale bar = 5 μm. *B–C*, the efficiency of siAjNLR-new in coelomocytes was determined using qPCR and western blotting analysis. The protein expression pattern was digitalized using Image J software by scanning the western blotting bands. The relative expression levels of AjNLR-new/β-Actin were expressed as the mean ± SD, and the value of the NC was set as one. *D*, flow cytometry was performed after knockdown of AjNLR-new to investigate the phagocytic activity of coelomocytes against AJ01. *E*, to detect the difference in the number of intracellular bacteria after siAjNLR-new. After *V*. *splendidus* was coincubated with coelomocytes, the unattached bacterial were washed away. The *V*. *splendidus* internalized into the coelomocytes were counted after killing the external *V*. *splendidus* with gentamicin, lysing the coelomocytes with Triton X-100. The immunoblot data are representative images from one of three independent experiments. n = 3 independent experiments (*A*) with similar results. Nuclei were stained with DAPI (*blue*). Student’s *t* test (*B*) were used for statistical analysis. Data are expressed as mean ± SD of 3 independent experiments (*B*), ∗∗*p* < 0.01.

AjNLRC4, composed of an Ig domain and a NACHT domain, has been shown to regulate the phagocytosis of AJ01 (25). To determine whether the AjNLR-new can regulate the phagocytosis of AJ01, an immunocytochemical assay is performed to detect the colocalization of AjNLR-new and AJ01. We observe that AjNLR-new colocalized with FITC-labeled AJ01, and this colocalization is accompanied by the cellular uptake of AJ01 at different time points (Fig. 3*A*). Afterward, the phagocytic activity of coelomocytes against AJ01 is further investigated, and the results showed that approximately 14.76% of coelomocytes could phagocytose AJ01 (Fig. 3*D*). Knockdown of AjNLR-new expression by siRNA transfection (Fig. 3, *B* and *C*) also significantly decreases phagocytic activity (Fig. 3*D*). The amount of intracellular AJ01 was significantly reduced (Fig. 3*E*).

### AjFidgetin serves as a downstream adaptor protein of AjNLR-new

**Figure 4:**
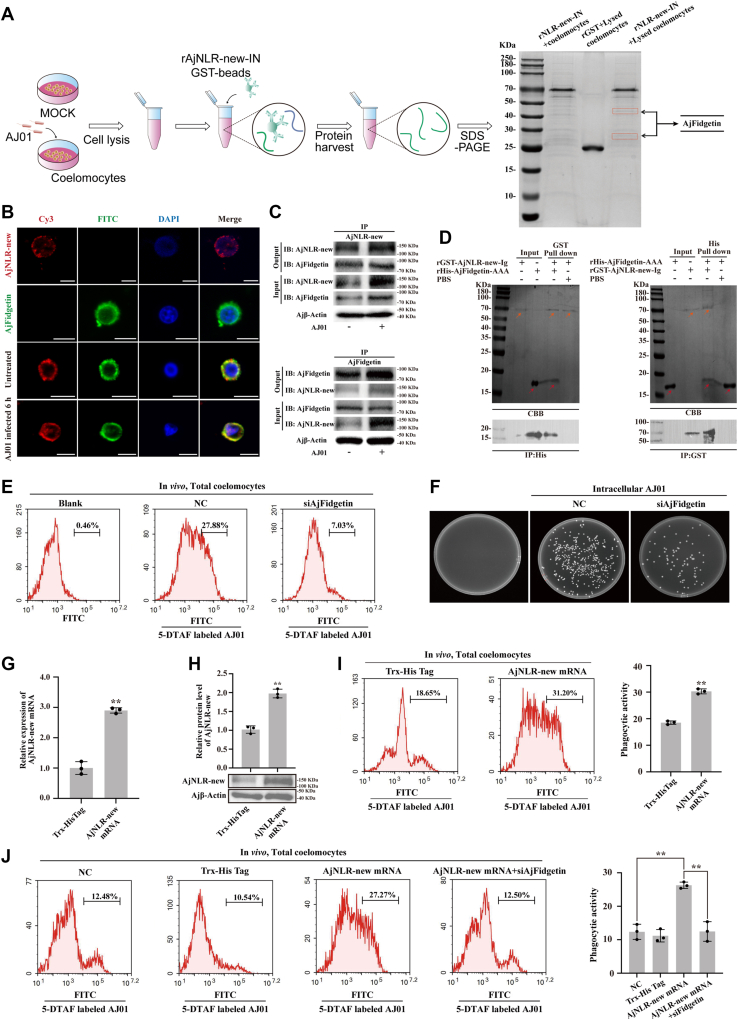
**AjNLR new regulates phagocytosis through microtubule-severing proteins AjFidgetin**. *A*, schematic diagram to detect AjNLR-new interactome by precipitation and mass spectrometry. Identification of the protein bound to AjNLR-new by mass spectrometry. The protein was identified to be AjFidgetin. *B-D*, to define the interaction between AjNLR-new and AjFidgetin. FITC-AjFidgetin was found to co-localize with Cy3-AjNLR-new by immunofluorescence (*B*) (Scale bar = 5 μm), and the interaction between AjNLR-new with AjFidgetin in *vivo* was analyzed by co-immunoprecipitation (Co-IP). Coelomocyte lysates before and after AJ01 infection were subjected to immunoprecipitation using AjNLR-new or AjFidgetin antibody (IP). The immunoprecipitated products were then analyzed by immunoblotting with AjFidgetin and AjNLR-new antibodies separately as the output. Coelomocytes lysates were used as input controls (*C*), and AjFidgetin and AjNLR-new were generated in *vitro*, and their interactions were further validated by using pull-down assays (*D*). *E*, flow cytometry was performed after knockdown of AjFidgetin to investigate the phagocytic activity of coelomocytes against AJ01. *F*, to detect the difference in the number of intracellular bacteria after siAjFidgetin. After *V*. *splendidus* was coincubated with coelomocytes, the unattached bacterial were washed away. The *V*. *splendidus* internalized into the coelomocytes were counted after killing the external *V*. *splendidus* with gentamicin, lysing the coelomocytes with Triton X-100. *G–H*, the efficiency of overexpression AjNLR-new in coelomocytes was determined using qPCR and western blotting analysis. The protein expression pattern was digitalized using Image *J* software by scanning the western blotting bands. The relative expression levels of AjNLR-new/β-Actin were expressed as the mean ± SD, and the value of the Trx-His Tag was set as one. *I–J*, flow cytometry was performed after overexpressed AjNLR-new and knockdown of AjFidgetin to investigate the phagocytic activity of coelomocytes against AJ01. The immunoblot data are representative images from one of three independent experiments. n = 3 independent experiments (*B*) with similar results. Nuclei were stained with DAPI (*blue*). Student’s *t* test (*G*, *I*, *J*) were used for statistical analysis. Data are expressed as mean ± SD of 3 independent experiments (*G*, *I*, *J*), ∗∗*p* < 0.01.

Membrane receptor-mediated phagocytosis depends on the interaction between its intracellular domain and downstream adaptor protein (29, 30). To explore the mechanism that underlies AjNLR-new-mediated phagocytosis, proteins that potentially interact with AjNLR-new-IN are identified by pull-down assays and MS/MS analysis. Among the analyzed proteins, Fidgetin (PIK44146.1) was identified as a possible AjNLR-new-IN interacting protein (Fig. 4*A*) (Fig. S2, *A* and *B*). The expression patterns of AjFidgetin mRNA in different tissues of *A*. *japonicus* were analyzed by real-time PCR and the Ajβ-actin gene was served as an internal reference. As shown in Figure S2*C*, AjFidgetin was commonly expressed in all detected tissues. After immersion infection with AJ01 in *vivo*, the expression level of AjFidgetin in coelomocytes was significantly increased (Fig. S2*D*). Immunofluorescence analysis indicated that AjNLR-new could colocalize with AjFidgetin in coelomocytes, and the signal was enhanced after AJ01 infection (Fig. 4*B*). We then validated the interaction between AjNLR-new and AjFidgetin by Co-IP assay. The results showed a clear interaction between AjNLR-new and AjFidgetin, which was stronger after AJ01 infection (Fig. 4*C*). Furthermore, reverse GST and His pull-down assays also confirm AjNLR-new-IN bound to AjFidgetin-AAA domain (Fig. 4*D*).

### AjFidgetin is required for AjNLR-new-mediated phagocytosis

Fidgetin, a microtubule-severing protein, regulates cell proliferation, migration, and differentiation by regulating the microtubule network (31). However, whether Fidgetin is involved in regulating phagocytosis is still unknown. In order to investigate whether AjFidgetin plays a regulatory role in the phagocytosis of AJ01 by coelomocytes, we performed RNAi-mediated AjFidgetin knockdown by using a specific siRNA (Table S1). Protein and mRNA expression levels of AjFidgetin were significantly downregulated after AjFidgetin siRNA transfection (Fig. S2, *E* and *F*). Meanwhile, the phagocytic activity of AJ01 and the number of intracellular AJ01 were both significantly reduced (Fig. 4, *E* and *F*). To investigate whether AjFidgetin is required for the AjNLR-new-mediated phagocytosis of AJ01, the expression level of AjNLR-new is overexpressed by mRNA injection (Fig. 4, *G* and *H*). Under this condition, coelomocyte phagocytic activity is significantly increased (Fig. 4*I*), while injection of AjFidgetin siRNA (Since there is currently no effective inhibitor that directly acts on Fidgetin) inhibited the phagocytic activity mediated by AjNLR-new overexpression (Fig. 4*J*).

### AjFidgetin mediates AJ01 phagocytosis by recruiting and severing microtubules to regulate microtubule depolymerization

**Figure 5:**
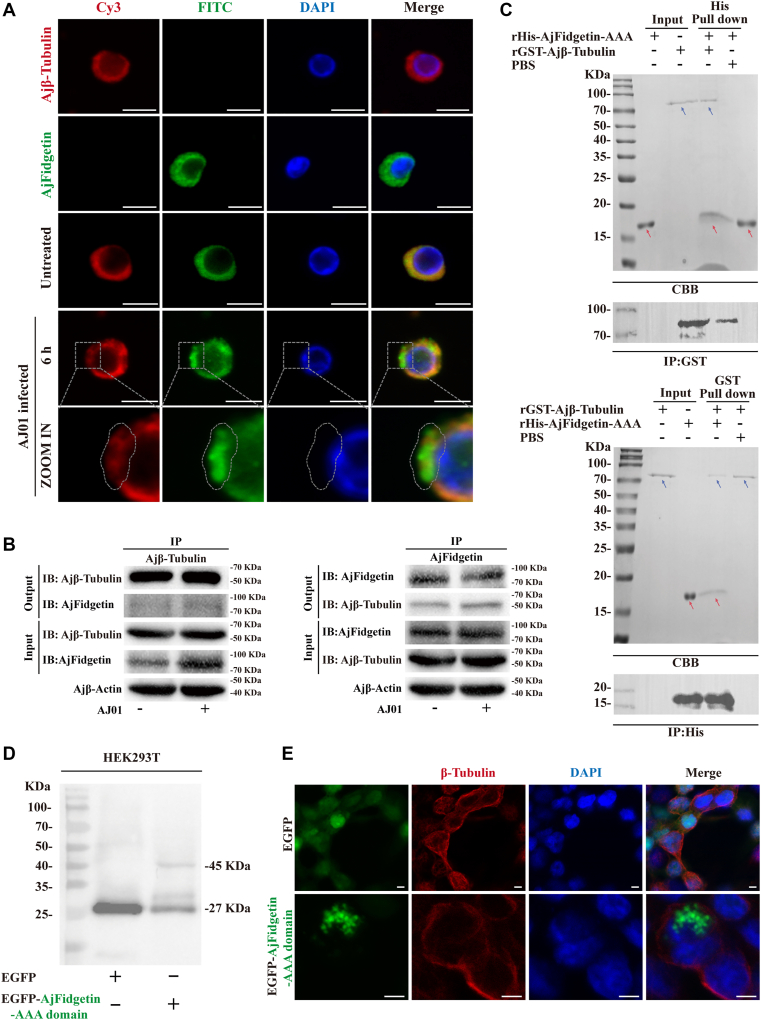
**AjFidgetin interacts with Ajβ-Tubulin *via* AAA domain**. *A–C*, to define the interaction between AjNLR-new and Ajβ-Tubulin. FITC-AjFidgetin was found to co-localize with Cy3-Ajβ-Tubulin by immunofluorescence (*A*) (Scale bar = 5 μm), and the interaction between Ajβ-Tubulin with AjFidgetin in *vivo* was analyzed by co-immunoprecipitation (Co-IP). Coelomocyte lysates before and after AJ01 infection were subjected to immunoprecipitation using AjFidgetin or β-Tubulin antibody (IP). The immunoprecipitated products were then analyzed by immunoblotting with AjFidgetin and β-Tubulin antibodies separately as the output. Coelomocytes lysates were used as input controls (*B*), and AjFidgetin and Ajβ-Tubulin were generated in *vitro*, and their interactions were further validated by using pull-down assays (*C*). *D*, Western blot analysis was used to detect the transfection efficiency of EGFP-AjFidgetin-AAA in 293T. *E*, EGFP-AjFidgetin-AAA was found to co-localize with β-Tubulin by immunofluorescence in 293T. Scale bar = 5 μm. The immunoblot data are representative images from one of three independent experiments. *n* = 3 independent experiments (*A*, *E*) with similar results. Nuclei were stained with DAPI (*blue*).

After determining that AjNLR-new regulates phagocytosis by recruiting AjFidgetin, it is still unknown how AjFidgetin mediates the phagocytosis of AJ01. The regulation of microtubule cytoskeleton by Fidgetin depends on its recognition and severing of microtubules (31). However, it is not clear whether AjFidgetin interacts with tubulin in *A*. *japonicus*. The interaction between AjFidgetin and Tubulin was analyzed using immunofluorescence, Co-IP and Pull-down experiments. Immunofluorescence analysis showed that AjFidgetin can co-localize with tubulin, and the complex signal was significantly enhanced after AJ01 infection (Fig. 5*A*), which was consistent with increased AjFidgetin-interactive β-tubulin intensity by AjFidgetin antibody as a bait and increased AjFidgetin intensity by β-tubulin antibody for Co-IP analysis (Fig. 5*B*). Furthermore, GST and His pull-down assays confirmed that AjFidgetin binds to tubulin *via* its AAA domain (Fig. 5*C*). Furthermore, immunofluorescence analysis of coelomocytes revealed that AJ01 infection led to substantial AjFidgetin aggregation and dissipation of the microtubule network at these aggregation sites. This phenomenon is consistent with the microtubule depolymerization observed in 293T cells transfected with AjFidgetin (Fig. 5, *D* and *E*). This indicates that AJ01 infection causes AjFidgetin to bind to and cut microtubules, causing microtubule depolymerization.

**Figure 6:**
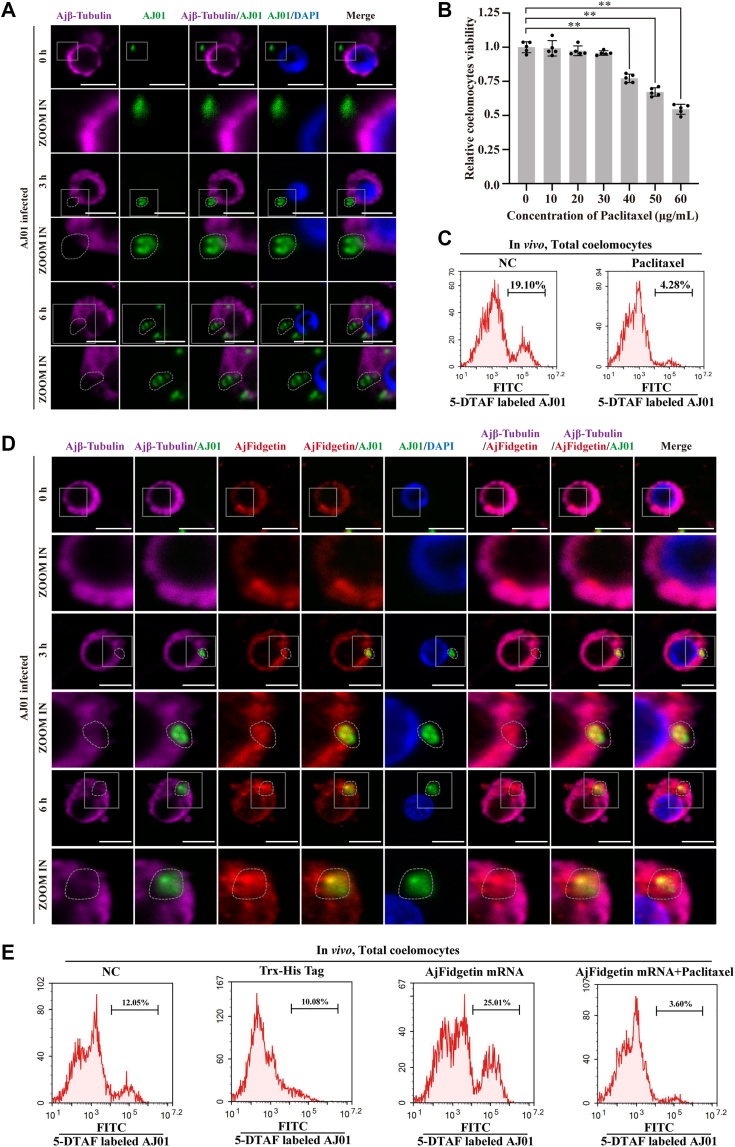
**The phagocytosis of AJ01 is closely related to the sever of microtubules**. *A*, immunocytochemistry was used to detect the colocalization of Ajβ-Tubulin and 5-DTAF-labeled AJ01 in coelomocytes. The coelomocytes were collected at different time points (0, 3, and 6 h) post-AJ01 injection. Scale bar = 5 μm. *B*, the effect of paclitaxel on the cell viability of sea cucumbers. Sea cucumber coelomocytes were treated with increasing concentrations of paclitaxel for 3 h, and cell viability was calculated. *C*, flow cytometry was performed after paclitaxel treatment to investigate the phagocytic activity of AJ01. *D*, immunocytochemistry was used to detect the colocalization of AjFidgetin, Ajβ-Tubulin and 5-DTAF-labeled AJ01 in coelomocytes. Scale bar = 5 μm. *E*, flow cytometry was performed after knockdown of AjFidgetin and paclitaxel treatment to investigate the phagocytic activity of coelomocytes against AJ01. The immunoblot data are representative images from one of three independent experiments. n = 3 independent experiments (*A*, *D*) with similar results. Nuclei were stained with DAPI (*blue*). Student’s *t* test (*B*) were used for statistical analysis. Data are expressed as mean ± SD of 5 independent experiments (*B*), ∗∗*p* < 0.01.

However, it is still unknown whether the phenomenon of microtubule depolymerization caused by AjFidgetin binding and severing is related to the phagocytosis of AJ01. Furthermore, it is unclear whether microtubules are involved in the regulation of AJ01 phagocytosis. Therefore, we first observed the morphology of microtubules during AJ01 endocytosis and the localization of microtubules and FITC-labeled AJ01. The results showed that the microtubule cytoskeleton of cells that did not undergo phagocytosis was more aggregated, while those that microtubule cytoskeleton of cells undergoing phagocytosis was depolymerized and decreased microtubule fluorescence intensity. Even more surprising, microtubules around the intracellular AJ01 were significantly depolymerized, forming vacuoles surrounding it (Fig. 6*A*). Subsequently, paclitaxel, a microtubule stabilizer, was used to treat coelomocytes to confirm that microtubule depolymerization is essential for AJ01 phagocytosis. Treatment of each sea cucumber with paclitaxel at concentrations below 30 μM did not affect coelomocyte viability (Fig. 6*B*). Therefore, coelomocytes were treated with 30 μM paclitaxel and then infected with FITC-labeled AJ01 for 3 h. We observed a significant decrease in the phagocytic activity of coelomocytes AJ01 after paclitaxel treatment (Fig. 6*C*). This suggests that the microtubule network of coelomocytes relies on its dynamic instability to mediate the phagocytosis of AJ01. We further investigated whether microtubule depolymerization during phagocytosis is related to AjFidgetin-mediated microtubule cleavage. We observed morphological changes in AjFidgetin and the microtubule network during AJ01 internalization. Consistent with previous observations, AjFidgetin accumulated abundantly at the site of AJ01 entry, while the microtubule network depolymerized to form diffuse vacuoles (Fig. 6*D*). To further elucidate this regulatory pathway, we examined the phagocytic activity of AJ01 in the presence of AjFidgetin overexpression (Fig. S2, *G* and *H*) and treatment with paclitaxel. The results showed that the upregulation of phagocytic activity caused by AjFidgetin overexpression was significantly inhibited by paclitaxel (Fig. 6*E*).

### AjNLR-new recruits AjFidgetin to regulate microtubule depolymerization and mediate phagocytosis of AJ01

**Figure 7:**
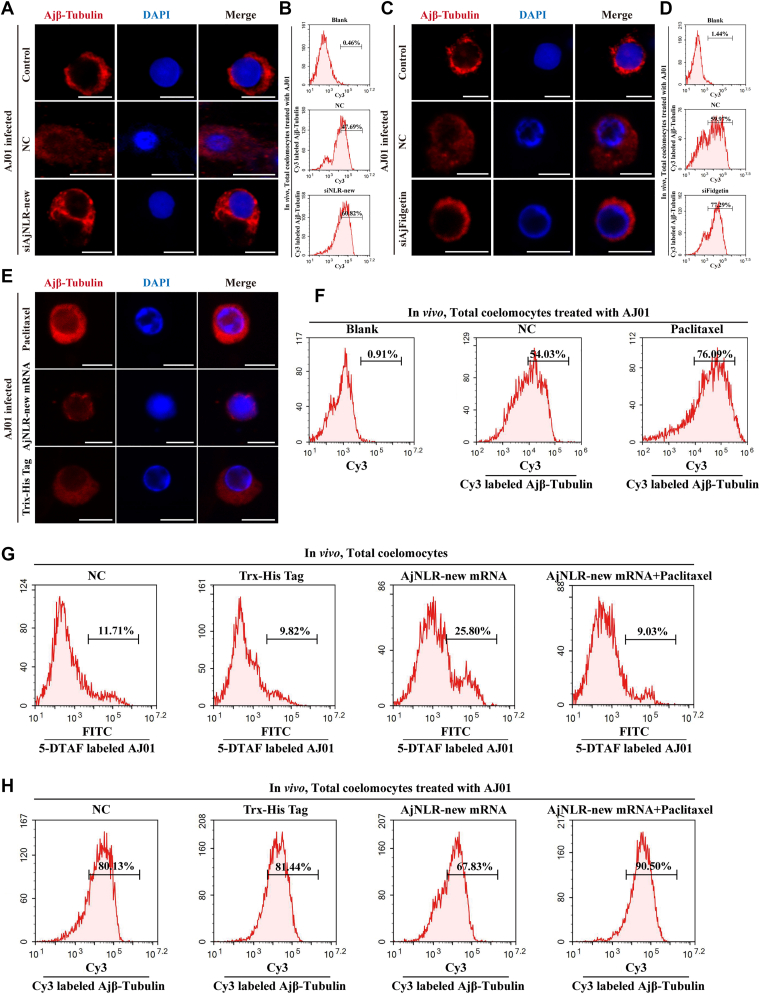
**AjNLR-new recruits AjFidgetin to mediate the dynamic instability of microtubules**. *A*, immunocytochemistry was performed after knockdown of AjNLR-new to investigate the depolymerization of microtubule cytoskeleton. *B*, flow cytometry was performed after knockdown of AjNLR-new to investigate the fluorescence intensity of microtubule cytoskeleton. *C*, immunocytochemistry was performed after knockdown of AjFidgetin to investigate the depolymerization of microtubule cytoskeleton. *D*, flow cytometry was performed after knockdown of AjFidgetin to investigate the fluorescence intensity of microtubule cytoskeleton. *E*, immunocytochemistry was performed after paclitaxel treatment to investigate the depolymerization of microtubule cytoskeleton. *F*, flow cytometry was performed after paclitaxel treatment to investigate the fluorescence intensity of microtubule cytoskeleton. *G*, flow cytometry was performed after knockdown of AjNLR-new and paclitaxel treatment to investigate the phagocytic activity of AJ01. *H*, flow cytometry was performed after knockdown of AjNLR-new and paclitaxel treatment to investigate the fluorescence intensity of microtubule cytoskeleton. The immunoblot data are representative images from one of three independent experiments. n = 3 independent experiments (*A*, *C*, *E*) with similar results. Nuclei were stained with DAPI (*blue*).

After clarifying that the phagocytosis of AJ01 by sea cucumber coelomocytes depends on microtubule depolymerization mediated by AjFidgetin, and that the regulation of phagocytosis by AjNLR-new depends on AjFidgetin, in order to further clarify the regulation of the microtubule skeleton by AjNLR-new and AjFidgetin, the expression of AjNLR-new and AjFidgetin were knocked down, respectively, and the morphological changes of microtubules during AJ01 infection were observed. The changes in fluorescence intensity of different forms of microtubules (the fluorescence intensity of polymerized microtubules will be higher) were detected by flow cytometry. Knockdown of AjNLR-new and AjFidgetin both resulted in more polymerization of microtubules and increased fluorescence intensity (Fig. 7, *A*–*D*). While it is clear that AjNLR-new and AjFidgetin promote microtubule depolymerization, it remains unclear whether AJ01 phagocytosis mediated by AjNLR-new recruiting AjFidgetin depends on microtubule disaggregation. We first examined the effect of the microtubule stabilizer paclitaxel on microtubule morphology. Treatment with the microtubule stabilizer paclitaxel significantly inhibited microtubules disassembly and enhanced the fluorescence intensity of microtubules (Fig. 7, *E* and *F*). We then examined changes in phagocytic activity and microtubule fluorescence intensity after 3 h of treatment with the microtubule stabilizer paclitaxel after AjNLR-new overexpression. The results showed that AjNLR-new overexpression significantly promoted phagocytic activity and microtubule depolymerization, which was inhibited by treatment with the microtubule stabilizer paclitaxel (Fig. 7, *G* and *H*).

### AjNLR-new recruits AjFidgetin to participate in multiple endocytic pathways and promote cytoskeletal rearrangement to regulate the phagocytosis of AJ01

**Figure 8:**
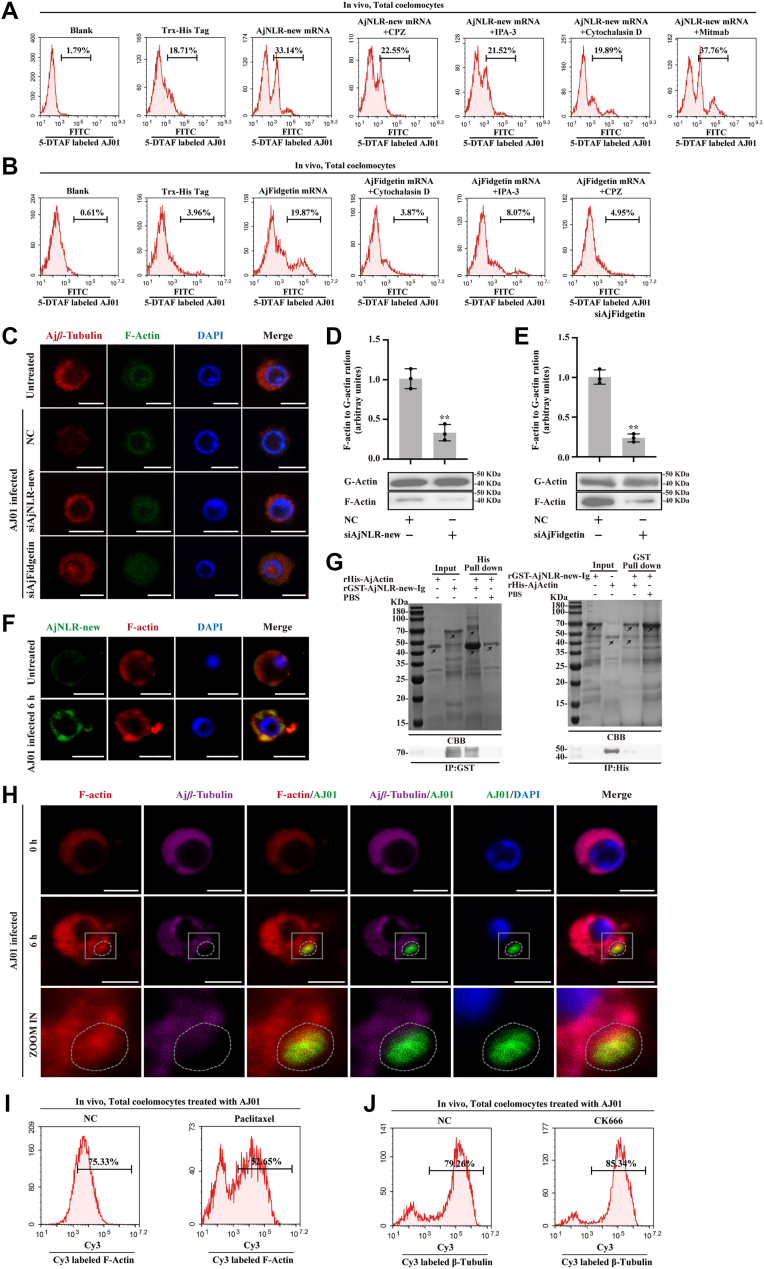
**AjNLR-new induces microtubule depolymerization, thereby promoting actin polymerization to regulate phagocytosis of AJ01**. *A–B*, after the overexpression of AjNLR-new or AjFidgetin, four specific inhibitors (chlorpromazine, CPZ, clathrin-dependent endocytic pathway inhibitor; IPA-3, macropinocytosis pathway inhibitor; cytochalasin D, actin-dependent endocytic pathway inhibitor and mitmab, dynamin-dependent endocytic pathway inhibitor) were added, and then, flow cytometry was performed to detect the phagocytic activity. *C*, immunocytochemistry was performed after knockdown of AjNLR-new or AjFidgetin to investigate the depolymerization of microtubules and microfilaments cytoskeleton. *D–E*, Western blotting revealed the change in the ratio F-actin/G-actin after treatment with siAjNLR-new or siAjFidgetin. (*F*) To define the interaction between AjNLR-new and F-actin. FITC-AjNLR-new was found to co-localize with Cy3-F-actin by immunofluorescence (Scale bar = 5 μm). *G*, the interactions of AjNLR-new and Actin was validated by GST/His pull-down assays. *H*, immunocytochemistry was used to detect the colocalization of AjNLR-new, F-actin and 5-DTAF-labeled AJ01 in coelomocytes. Scale bar = 5 μm. *I*, flow cytometry was performed after paclitaxel treatment to investigate the fluorescence intensity of microfilament cytoskeleton. *J*, flow cytometry was performed after CK666 treatment to investigate the fluorescence intensity of microtubule cytoskeleton. The immunoblot data are representative images from one of three independent experiments. n = 3 independent experiments (*C*, *F*, *H*) with similar results. Nuclei were stained with DAPI (*blue*). Student’s *t* test (*D*, *E*) were used for statistical analysis. Data are expressed as mean ± SD of 3 independent experiments (*D*, *E*), ∗∗*p* < 0.01.

Previous work in our laboratory has demonstrated that phagocytosis of AJ01 depends on clathrin-, macropinocytosis-, actin-, and dynein-mediated phagocytic pathways (25). To definitively clarify the phagocytic pathway regulated by AjNLR-new recruitment of AjFidgetin, we overexpressed AjNLR-new and then treated coelomocytes overexpressing AjNLR-new with four phagocytic pathway inhibitors: Cytochalasin D (actin-dependent endocytic pathway inhibitor), Chlorpromazine (CPZ, clathrindependent endocytic pathway inhibitor), IPA-3 (macropinocytosis pathway inhibitor), and Mitmab (dynamin-dependent endocytic pathway inhibitor). Flow cytometry was then used to assess the phagocytic activity of the cells. The results showed that the enhanced phagocytic activity of cells overexpressing AjNLR-new could be blocked by cytochalasin D, chlorpromazine, and IPA-3, while there was no change in the Mitmab-treated group (Fig. 8*A*). This suggests that AjNLR-new regulates phagocytosis through the actin, clathrin, and macropinocytosis pathways. Further investigation was then conducted to determine which phagocytic pathway AjFidgetin, a downstream target of AjNLR-new recruitment, mediates. The same method was used to detect the AjFidgetin-mediated phagocytosis pathway. The results showed that the AjFidgetin-mediated phagocytosis pathway was consistent with the AjNLR-new-mediated phagocytosis pathway (Fig. 8*B*). Notably, the actin-mediated phagocytosis regulatory pathway was the most inhibited.

To analyze the mechanism by which AjNLR-New–AjFidgetin axis regulates the microfilament cytoskeleton, the effects of AjNLR-New and AjFidgetin interference on microfilament cytoskeleton were examined. Knockdown of AjNLR-new and AjFidgetin expression depolymerizes F-actin, which inhibits the maintenance of its original morphology (Fig. 8*C*), whilst simultaneously suppressing the conversion of F-actin to G-actin (Fig. 8, *D* and *E*). So how does AjNLR-New affect microfilament cytoskeleton rearrangement? It has been reported that proteins containing intracellular Ig domains could directly in anchor to the cytoskeleton components. Based on our lab previous findings that phagocytosis in AJ01 is directly related to the microfilament cytoskeleton (25). Therefore, the co-localization of AjNLR-new and actin was examined, and it was found that AJ01 infection promotes the co-localization of AjNLR-new and F-actin (Fig. 8*F*). Subsequently, the interaction between the intracellular Ig domain of AjNLR-new and β-Actin was clarified through GST/His-Pull down assay (Fig. 8*G*). How then does AjFidgetin, which is recruited by AjNLR-new and participates in phagocytosis regulation by severing microtubules, participate in actin-dependent endocytosis? Therefore, we hypothesize that there may be a potential regulatory relationship between the microtubule cytoskeleton and the microfilament cytoskeleton. We analyzed the subcellular localization of AJ01, Tubulin and F-actin during the phagocytosis of AJ01 by coelomocytes. In addition to the phenomenon of microtubules depolymerization and forming cavitation at the AJ01 phagocytosis site, consistent with the previous results (Fig. 6*A*), the F-actin are co-located with AJ01 and aggregate and encapsulate AJ01 at the cavitation sites formed by microtubule dissipation (Fig. 8*H*). As regulation of microtubules by AjFidgetin could be blocked by microtubule stabilizers (paclitaxel), and knockdown the AjFidgetin significantly inhibits microfilament polymerization, we treated coelomocytes with microtubule stabilizers and subsequently examined microfilament polymerization. The results showed that the microtubule stabilizer could inhibit the polymerization of microfilaments (Fig. 8*I*). However, treatment of coelomocytes with the microfilament polymerization inhibitor CK666 did not affect microtubule disintegration (Fig. 8*J*), indicating that microfilament polymerization is regulated by microtubule dynamic instability. Therefore, AjNLR-new induces microtubule severing by recruiting fidgetin, thereby promoting microfilament polymerization and mediating phagocytosis by AJ01.

### AjNLR-new mediates the clearance of phagocytosed AJ01 *via* the lysosomal pathway

**Figure 9:**
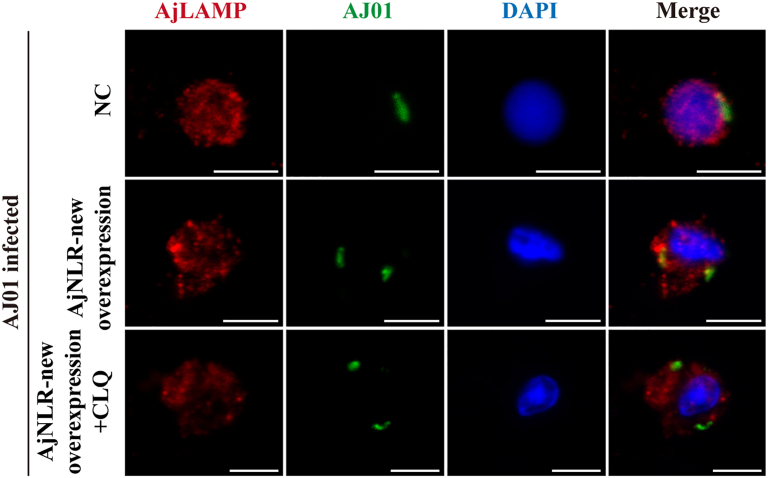
**Endocytosed *V*. *splendidus* is further cleared *via* lysosome degradation**. Colocalization of ingested AJ01and lysosomes. Twenty-four hours after the injection of 40 μM CLQ, 5-DTAF-labeled AJ01 was injected into sea cucumbers. Scale bar = 5 μm. The immunoblot data are representative images from one of three independent experiments. n = 3 independent experiments with similar results. Nuclei were stained with DAPI (*blue*).

The lysosomal degradation pathway is the main and core pathway for the processing and elimination of phagocytic substances. Pathogens that enter the cell form phagosomes, which fuse with lysosomes to form phagolysosomes. The lysosomes are then activated and begin to efficiently decompose the phagocytosed pathogens. To demonstrate whether AJ01 phagocytosed by AjNLR-new is cleared through the lysosomal pathway, we detected the colocalization of FITC-labeled AJ01 and lysosomes after injection of CQ (chloroquine, a lysosomal fusion inhibitor) under AjNLR-new overexpression conditions. The results showed that the co-localization of FITC-labeled AJ01 and lysosomes was significantly enhanced in the AjNLR-new overexpression group compared with the NC group, while the co-localization of FITC-labeled AJ01 and lysosomes was significantly reduced in the AjNLR-new overexpression + CQ treatment group compared with the AjNLR-new overexpression group (Fig. 9).

## Discussion

**Figure 10:**
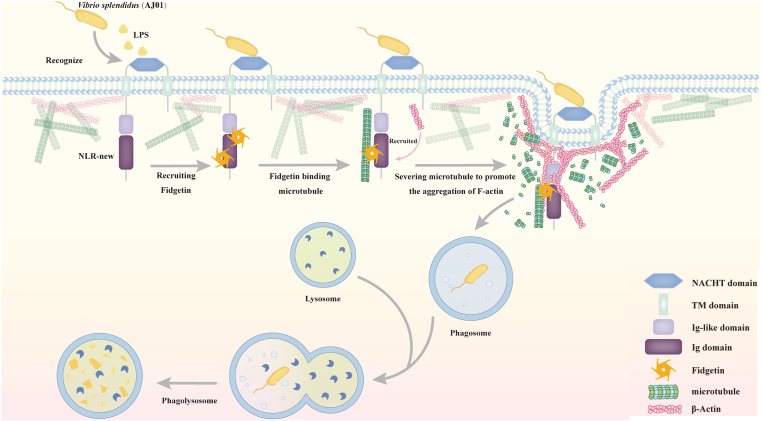
**Schematic of the role of AjNLR-new in promoting *V*. *splendidus* endocytosis as a transmembrane receptor**. *V*. *splendidus* binds to the extracellular domains of AjNLR-new and activates receptor-mediated endocytosis. The intracellular domain of AjNLR-new interacts with AjFidgetin, the recruited AjFidgetin binds to and severs microtubules, leading to the depolymerization of the microtubule cytoskeleton. and forms vacuoles around the phagocytosed *V*. *splendidus*, thereby facilitating F-actin polymerization at the vacuole sites. Finally, *V*. *splendidus* is degraded in coelomocyte phagolysosomes, which effectively restricts *V*. *splendidus* infection in sea cucumbers.

NLRs encoding NACHT modules in mammals have been shown to play a crucial role in resisting invasion by foreign pathogens and possess potent antibacterial properties. A large number of NLR-associated proteins with novel combinations of NACHT-domains have emerged in invertebrates, including unique combinations of TM domain and NACHT-domains. Currently, research on NLR-related proteins with TM domain is still lacking. Our lab has previously identified a novel transmembrane NLR with an extracellular Ig domain and an intracellular NACHT domain. A comparison of its immune regulatory pathways with those of vertebrate NLRs has revealed similarities and differences (25, 32). Here, we demonstrate that a novel NLR (AjNLR-new) with an extracellular NACHT-domain and an intracellular Ig-domain acts as a receptor for *V*. *splendidus*. This receptor regulates microtubule depolymerization by recruiting microtubule-severing proteins and promotes microfilament polymerization, ultimately mediating the clearance of phagocytosed AJ01 *via* the lysosomal pathway (Fig. 10). This is the first report on NLR with an extracellular NACHT domain mediating the regulation of innate immunity.

The NACHT domain, a classic shared domain of NLRs, has been reported to be widespread and numerous across species. In addition to being found in sea urchins, corals, and sponges, numerous novel genes containing NACHT domains have even been identified in bacteria and fungi, suggesting that bacterial NACHT proteins are related to eukaryotic NLRs. Current research suggests that bacteria encode the largest diversity of NACHT module sequences compared with other superkingdoms, which suggests that this protein module first evolved in bacteria before being acquired into the genomes of eukaryotes, and these genes have horizontally transferred from prokaryotes to eukaryotes on multiple occasions (23). The N-terminal regions of bacterial NACHT proteins encode many enzymatic domains that have previously been associated with biological conflict, including nucleases (RNases and DNases), peptidases, nucleotide signal-generating or degrading domains, and NAD^+^-targeting enzymes (toll/interleukin receptor [TIR] and Sirtuin) (33, 34, 35, 36). The unique combination of transmembrane domain, Ig domain and NACHT domain found in sea cucumbers (*A*. *japonicus*) is another strong evidence for the diversity of N-terminal effector domains in the early ancestors of NLR-related proteins. It is worth noting that there are multiple proteins with extracellular NACHT domains in bacteria, such as WP_100447268.1 from *G*. *xiaoerkulensis* and WP_070225684.1 from *Vibrio parahaemolyticus*. Their NACHT domains are located after the two transmembrane domains at the N- terminal, and analysis of the intracellular and extracellular domain showed that their NACHT domains are located extracellularly; bNACHT42 from *Klebsiella variicola* (K.v.) also has two transmembrane domains at the N-terminus, and is composed of a BPs domain located in the intracellular domain and a NACHT domain located extracellularly (23). The structural domain composition of these NACHT-localized extracellular bacterial NLR-related proteins is highly similar to that of AjNLR-new. This suggests that AjNLR-New may have originated from bacterial NLR-related proteins, similar to other eukaryotes, as a product of the pathogen-host arms race. Moreover, because the NACHT domains have strong homotypic domain interaction ability, AjNLR-new is likely to be the key receptor for NACHT extracellular NLR-related proteins in bacteria. More importantly, AjNLR-new relies on its extracellular NACHT domain to directly recognize and bind to various PAMPs and bacteria. Traditionally, the NACHT domain is primarily responsible for nucleotide-dependent molecular switch functions rather than directly recognizing PAMPs and DAMPs. Previous studies have shown that hydrophobic interactions play an important role in the LPS-receptor binding process. For example, TLR4's recognition of LPS depends on the hydrophobic region of its extracellular segment (37). Even Caspase-4/5/11's recognition of LPS also depends on the hydrophobicity of the CARD domain (38). Interestingly, AjNLR-new-NACHT also has multiple hydrophobic regions. These hydrophobic regions may be the key factor in the NACHT domain's ability to recognize PAMPs.

Classical NLRs activate upon pathogen recognition and binding, regulating pyroptosis and inflammation. However, in this study, AjNLR-new regulates phagocytosis. This process represents a novel NLR-mediated mechanism of resistance to pathogen infection. This mechanism is completely different from the traditional mechanism by which NLRs resist pathogenic infection, and this new mechanism might be the result of the combination of the N-terminal Ig domain and the NACHT domain. Some studies have shown that domain architectures are largely conserved (39). However, a more recent study indicates that domain architecture reinvention is a more common phenomenon than previously thought (40). NACHT is a representative of parallel evolution of innate immune receptors. The NACHT domain contains protein–protein interaction domains that contribute to signal transduction. Not only the NACHT domains themselves but also the domains they associate with, such as Death, CARD, and Ig domains. However, only a small fraction of the possible domain combinations actually exist in nature, which suggests that domain architectures are under strong evolutionary selection (41). This novel combination of an Ig domain and a NACHT domain in *A*. *japonicus* could serve as another example of domain architecture reinvention. Previous studies have shown that the function of NLRs is highly dependent on their N-terminal effector domains. The N-terminal effector structure of AjNLR-new is the intracellular Ig domain. Proteins that are located in the cytoplasm and contain Ig domains, such as myosin-binding protein C (cMyBP-C), titin, myosin (Myotiltin), palladin, and myopalladin, all directly or indirectly anchor to the cytoskeleton or cytoskeleton-related proteins through their Ig domains, thereby playing a key role in the dynamic regulation of the cytoskeleton. This is consistent with our findings that AjNLR-new mediates the phagocytosis of *V*. *splendidus* by regulating the rearrangement of the cytoskeleton through the intracellular Ig domain. AjFidgetin, as the downstream adaptor protein of AjNLR-new, interacts with the Ig domain, which may be dependent on the conserved *β*-sandwich structure of the Ig domain (42), which is crucial for the interaction of the Ig domain with multiple proteins (43). AjFidgetin contains a conserved microtubule-binding and severing key domain, the AAA domain, which can cleave microtubules like vertebrate Fidgetin, causing the microtubules to become diffuse. In our laboratory's preliminary research, we observed extensive microfilament polymerization at the phagocytosed sites of *Vibrio splendens* (25). However, this study reveals that microtubules depolymerize at these sites to form vacuoles. This phenomenon of microfilament polymerization at the site of microtubule dissipation may suggest an antagonistic interaction between microfilaments and microtubules during engulfment. Interestingly, we also demonstrated that AjNLR-new can directly interact with the cytoskeletal component actin through the Ig domain.which explains why AjNLR-new not only regulates the dynamic instability of microtubules but also promotes the conversion of G-actin to F-actin, thereby achieving dual regulatory functions of the microtubule network and the microfilament network. This is consistent with the phenomenon of microfilament polymerization at the site of microtubule dissipation observed in the early stages of macrophage phagocytosis of microspheres reported by Yoshika Seta *et al*. (44). Not only that, but they also found that the destabilization of the microtubule triggered membrane ruffling, and that the inhibitory effect of microtubules on actin network remodeling and membrane ruffling. Pineau *et al*. (45) also reported a similar regulatory effect of microtubules. They demonstrated that microtubules and the GEF–H1–RhoA axis had an inhibitory effect on actin polymerization at the lymphocyte–antigen contact site. In summary, we identified the first extracellular NLR-related protein with a NACHT domain from sea cucumbers and analyzed its new mechanism of mediating the phagocytosis of *V*. *splendens* through dual regulation of microtubule network and microfilament network, providing new insights into the functional evolution of invertebrate NLRs.

## Experimental procedures

### Ethics statement

The sea cucumbers used in this work were commercially cultured animals, and all experiments were conducted in accordance with the recommendations in the Guide for the Care and Use of Laboratory Animals of the National Institutes of Health. The study protocol was approved by the Experimental Animal Ethics Committee of Ningbo University, China.

### Animals

Healthy adult sea cucumbers (*A*. *japonicus*) weighing 125 ± 15 g were collected from the Dalian Pacific Aquaculture Company (Dalian, China) and temporarily maintained in 30 L of aerated natural seawater (salinity of 28 ± 1, temperature of 16 ± 1 °C) for two days. Randomly select sea cucumbers of uniform size for the following experiment.

### Bacterial immersion infection

The *V*. *splendidus*-related strain AJ01 used in this study was isolated from diseased *A*. *japonicus*. The bacterium was identified by 16S rDNA sequence analysis, and its pathogenicity was determined in our previous study (46, 47). AJ01 was cultured at 28 °C in 2216E medium consisting of 5 g/L tryptone, 1 g/L yeast extract and 0.01 g/L FePO4 in aged seawater. For immersion infection, AJ01 cells were collected by centrifugation at 5000×*g* for 10 min and resuspended in filtered seawater. Sea cucumbers were randomly divided into four tanks each containing 10 individuals. The three experimental groups were infected by immersion with live AJ01 at a final concentration of 1 × 10^7^, 1 × 10^8^, or 1 × 10^9^ CFU/ml. The untreated group served as the control. After immersion infection for 0, 3, 6, and 12 h, coelomic fluids were collected from each individual in each group, passed through a 200 mm sterile nylon mesh, and centrifuged at 800×*g* for 5 min. Three biological replicates were conducted for each sampling point and used for subsequent related experimental analysis.

### Recombinant expression and antiserum preparation

Use specific primers AjNLR-new-EX-F and AjNLR-new-EX-R (Table S1) to amplify the extracellular fragment of AjNLR-new. Purification of recombinant AjNLR-new-EX protein using Ni NTA Sepharose column (Roche) and dialysis at 4 °C to restore protein activity. Quantify the concentration of recombinant protein using the double sinnic acid method (Sangon Biotechnology, Shanghai). Similarly, the sequences of AjNLR-new-IN, AjFidgetin-AAA, and Ajβ-Tubulin (BAH79732.1) were amplified (primers listed in Table S1) and cloned into pET-28a^+^ and PGEX-4T-1 vectors to generate corresponding recombinant proteins for refolding. Purified GST tagged AjNLR-new-IN, His tagged AjFidgetin-AAA, and GST tagged Ajβ-Tubulin were used for further functional analysis. Prepare mouse serum against AjNLR-new and rabbit serum against AjFidgetin according to the previously reported method (48).

### PAMP binding assay

As mentioned earlier, the binding activity of PAMP was examined by enzyme-linked immunosorbent assay (ELISA) (24). Three types of PAMPs were used, namely LPS (*E*. *coli* 055: B5; Sigma), PGN (*Staphylococcus aureus*; Sigma) and MAN (*Saccharomyces cerevisiae*; Sigma), and another type of LPS was isolated from *V*. *splendidus* using an LPS extraction kit (Beibokit). Dissolve these PAMPs separately in carbonate bicarbonate buffer (50 mmol/L, pH 9.6), with a final concentration of 0.2 mg/L. Then, each PAMP solution was placed in a 96 well plate, with 30 μl per well, and incubated overnight at 4 °C. Wash three times with phosphate buffered saline (PBST, pH 7.2) containing 0.05% Tween-20. Add 200 μl of 5% BSA (prepared with PBS) to each well, incubate at 37 °C for 1 h, and then wash three times with PBST. Subsequently, 100 μl of different doses (0, 2.5, 5, 7.5, 10, 12.5, 15, 17.5 and 20 μg) of rAjNLR-new-EX protein were added to each well, incubated at 37 °C for 1 h, and washed twice with PBST. Add 100 μl of AP-labeled goat anti-mouse IgG (diluted 1:3000), incubate at 37 °C for 1 h, and then wash four times with PBST. Add 100 μl of PNPP chromogenic substrate reagent (Beijing Saici Biotechnology Co., Ltd) and incubate at room temperature in the dark for 30 min. Add 50 μl of 3 mol/L NaOH to each well to terminate the reaction and read at 450 nm using a microplate reader (Thermo Fisher Scientific). Use BSA of the same concentration (0, 2.5, 5, 7.5, 10, 12.5, 15, 17.5, and 20 μg) as a control, and Tris buffered saline (50 mmol/L Tris HCl, 50 mmol/L NaCl, pH 7.5) as a blank control. Each sample underwent five repeated analyses, and the data was represented as mean ± SD (n = 5).

### Microbial binding assay

Use G^+^ bacteria (*M*. *luteus*) and G^-^ bacteria (*V*. *harvey*, *V*. *parahemolyticus*, *V*. *splendidus* and *E*. *coli*) to test the binding activity of rAjNLR-new-EX (NACHT domain) and rAjNLR-new-IN (Ig-like-Ig domain). Bacteria were cultured overnight in the corresponding culture medium, centrifuged at a speed of 6000 g for 5 min, and the culture medium was removed to obtain bacteria. Wash four times with TBS, resuspend the bacteria in TBS, and adjust the bacterial density to A_600_ = 1.0. Take 200 μl and mix it with purified rAjNLR-new-EX and rAjNLR-new-IN (200 μl, 1 mg/ml), and incubate at room temperature for 1 h. Centrifuge at a speed of 6000 g for 5 min to remove the supernatant, collect bacteria, and then wash four times with TBS. Finally, after 12% SDS-PAGE, the protein in the gel was transferred to the nitrocellulose membrane, and the anti-His Ab (diluted in TBST containing 5% skimmed milk) and alkaline phosphatase coupled horse anti mouse IgG antibody (diluted in TBST containing 5% skimmed milk) were used as the first antibody and the second antibody for protein imprinting analysis.

### Microbial agglutination assay

Perform agglutination tests using *V*. *harvey*, *V*. *parahemolyticus*, *V*. *splendidus*, *E*. *coli*, and *M*. *luteus*. Harvest microorganisms in the logarithmic growth stage, centrifuge at 4000 g for 10 min, collect bacterial cells, wash once with 0.1 M NaHCO_3_ (pH 9.0), and stain with FITC (Sigma; 1 mg/ml in 0.1 M NaHCO_2_) at the corresponding culture temperature for 3 h (49). Wash FITC labeled microorganisms three times with PBS to eliminate all free FITC, and resuspend them in PBS at a density of A_600_ = 1.0. Mix 50 μl of microbial suspension with 50 μl of rAjNLR-new-EX (1 mg/ml) in the presence or absence of 10 mM CaCl_2_. Incubate the mixture at room temperature for about 2 h. Use BSA instead of rAjNLR-new-EX as the negative control. LPS (1 mg/ml) and PGN (1 mg/ml) were used for agglutination inhibition experiments to determine the binding specificity of G^-^bacteria and G^+^ bacteria, respectively. Ca^2+^ chelating agent EDTA (10 mM) was used as another control. Use a fluorescence microscope (Nikon) to analyze agglutination activity and image three independent fields of view.

### Immunocytochemical analysis

Drop the coelomocytes suspended in the culture medium onto a polylysine coated cell slide and culture for 6 h. Remove the culture medium, slowly add an appropriate amount of PBS, wash the slide three times, and remove non adherent cells. Add pre cooled 4% paraformaldehyde, fix the cells for 30 min, and wash them three times with PBS after completion. Add 0.5% Tritonx-100 (PBS configuration), permeabilize the cell membrane for 20 min, and wash three times with PBS after completion. Seal the cells with 5% bovine serum albumin (BSA, dissolved in PBS) for 30 min. Afterwards, add primary antibody (diluted in 5% bovine serum albumin) and incubate overnight at 4 °C. Remove the primary antibody and wash the cells three times with 0.1% PBST. Add the corresponding secondary antibody (diluted in 5% bovine serum albumin) and incubate at room temperature for 2 h. After completion, wash the cells three times with 0.1% PBST. Then stain with 4-6-diamidino-2-phenylindole (DAPI) at room temperature for 10 min. After washing the cells three times with 0.1% PBST, the glass slides were observed under a laser scanning confocal microscope (TCS SP2; Leica, Germany).

### Coelomocyte-binding activity of rAjNLR-new-EX and rAjNLR-new-IN

rAjNLR-new-EX and rAjNLR-new-IN were dialyzed in CFS and then injected into sea cucumber separately. Collect coelomocytes and culture them 1 h after injection. Detect whether the recombinant protein can bind to the surface of body cavity cells through the above-mentioned immunohistochemical method. One of the antibodies used was His tagged antibody, and FITC conjugated goat anti mouse IgG was used as the second antibody. Finally, the cells were observed under a laser scanning confocal microscope (TCS SP2; Leica).

### Co-localization of fluorescently labelled *V*. *splendidus* and lysosomes

Inject FITC labeled *V*. *splendidus* into sea cucumbers, collect the coelomocytes 3 h later and spread them on glass slides. Stain the lysosomes in coelomocytes with LysoTracker Red (Beyotime) and incubate at 16 °C for 3 h before observing them under a laser scanning confocal microscope.

### RNA interference assays

GenePharma designed and synthesized siRNA (Table S1) powder targeting AjNLR-new. Another type of siRNA (negative control, NC) with no specificity for any single gene in the transcriptome of *A*. *japonicus* was used as the negative control. Firstly, dissolve these siRNA dry powders in RNase-free water to produce a 20 μM working solution. Then, 10 μl of AjNLR-new siRNA and an equal volume of transfection reagent were mixed with 80 μl of phosphate-buffered saline (PBS) to prepare the transfection working solution. Inject 100 μl of the aforementioned transfection solution into each sea cucumber (weighing approximately 100 g). The control group was injected with NC siRNA under the same conditions. Collect coelomocytes for qPCR and Western blot analysis after 24 and 48 h respectively to evaluate interference efficiency. The treated and negative control groups were set up in triplicate. A similar method is used for AjFidgetin interference testing.

### AjNLR-new overexpression

The AjNLR-new open reading frame (ORF) was amplified using the AjNLR-new-ORF-F and AjNLR-new-ORF-R primers (Table S1). The PCR fragments were then ligated into the pET-32a^+^ vector, which contains a T7 promoter. Thereafter, the recombinant plasmid was used for mRNA synthesis and capping as previously described (27). The mRNA from the empty pET-32a^+^ vector was used as a control. Each sea cucumbers was injected with 300 μg mRNA and incubated for 24 h, and the overexpression efficiency was detected using AjNLR-new antibodies. Later, 5-DTAF-labeled *V*. *splendidus* was injected into the sea cucumbers for an additional 3 h. Thereafter, the coelomocytes were obtained by centrifugation at 800*g* for 5 min, washed and resuspended in PBS. Phagocytic activity, which is the ratio between phagocytic cells and total cells, was detected by flow cytometry. Extracellular fluorescence was quenched by adding 1 ml of 0.5% trypan blue to the cell suspension. The experiments were repeated three times. A similar method is used for AjFidgetin overexpression assay.

### Specific inhibitor assay

Pharmacological inhibitors were often used to investigate the endocytic mechanism responsible for the cellular uptake of particles (50). To explore the AjNLR-new mediated *V*. *splendidus* endocytosis pathway, we used four specific inhibitors, chlorpromazine (CPZ, clathrin-dependent endocytic pathway inhibitor, Sangon Biotech), IPA-3 (macropinocytosis pathway inhibitor, Aladdin), cytochalasin D (actin-dependent endocytic pathway inhibitor, Aladdin), and mitmab (dynamin-dependent endocytic pathway inhibitor, Aladdin). The appropriate working concentrations of four inhibitors that do not affect cell survival rate have been determined in the preliminary work. Treat the coelomocytes with these inhibitors for 24 h, while the control group was treated with DMSO alone. Afterwards, the coelomocytes were treated with the specified inhibitor concentration for 3 h, followed by injection of 100 μl 5DTAF-labeled *V*. *splendidus* (10^6^ CFU/ml) for 3 h. Measure phagocytic activity using flow cytometry.

### Flow cytometry assay phagocytic activity

Label *V*. *splendidus* with 5-DTAF (green) for 3 h, then collect by centrifugation at 5000×*g* for 10 min, wash twice with PBS, and finally suspend in PBS for sea cucumber injection experiments. Collect body cavity cells 3 h after injection and detect phagocytic activity using flow cytometry (ImageStreamX MarkII). A total of 10,000 cells were collected to quantify the percentage of phagocytic activity. Add 1 ml of 0.5% trypan blue to the cell suspension to quench extracellular fluorescence. The experiment was repeated three times.

### Internalization assay

*V*. *splendidus* were collected at OD_600_ = 1.0, washed with phosphate-buffered saline (PBS, 0.01 M sodium phosphate, 0.8% NaCl, pH 7.2) and resuspended in internalization medium (IM; lowglucose Dulbecco’s modified Eagle’s medium (Gibco) without 10% (v/v) fetal bovine serum (HighClone) and antibiotics). One milliliter of 10^6^ CFU/ml *V splendidus* suspension was separately added to coelomocyte monolayers in 24-well plates at a final concentration of approximately 10^6^ CFU/ml. After coincubation for 3 h, 6 h and 9 h, each well was washed with IM three times, and one portion of coelomocytes was detached by adding trypsin-like enzyme (TrypLe Express, Gibco) followed by Triton X-100 (0.025%) to release the intracellular bacteria. The other portion of coelomocytes was incubated for another 1.5 h in IM with 500 μg gentamicin (Gibco) per well to kill extracellular *V*. *splendidus*. The wells were washed three times, and trypsin-like enzyme and Triton X-100 were sequentially added to release the internalized bacteria. The number of bacteria in both lysates was determined by plate counts.

### Pull-down assay

Perform pull-down analysis to identify the AjFidgetin protein that interacts with recombinant AjNLR-new-IN. Use the primers listed in Table S1 to amplify the structural domain of AjNLR-new sequence, named AjNLR-new-IN. Connect AjNLR-new-IN to pGEX-4T-1 vector (GE Healthcare) and transform into *E*. *coli* Rosseta. Purification of recombinant expressed protein by affinity chromatography using GST affinity chromatography column material (GenScript). Perform GST pull-down analysis to identify AjNLR-new-IN interacting proteins. According to the manufacturer's instructions (C600913, Sangon Biotech), capture recombinant GST AjNLR-new-IN using GST SephaseTM affinity chromatography column material for GST pull-down assay. Incubate the protein extract of the column material and coelomic cells overnight at 4 °C. An equal volume of cell lysate was used as a control. Subsequently, wash the mixture three times with a binding/washing buffer solution that is 5 to 10 times the volume of the column material. Finally, the protein was eluted using GST elution buffer (10 mM reduced glutathione, 50 mM Tris HCl, pH 8.0). Analyze the eluted proteins through SDS-PAGE, compare these proteins with the control group samples, extract differential bands for further analysis by LC-MS/MS, to identify proteins that interact with AjNLR-new-IN.

Recombinant expression of AjNLR-new-IN interacting protein in *E*. *coli* using pET-28a^+^ expression vector. Purified GST labeled AjNLR-new-IN (200 μg) and His labeled AjNLR-new-IN interacting protein (200 μg) were incubated overnight with GST/His affinity chromatography column at 4 °C. Add equal amounts of His labeled AjNLR-new-IN interacting protein and GST labeled AjNLR-new-IN the next day, incubate at 4 °C for 6 h. Wash the resin 10 times with PBS and add GST elution buffer or His elution buffer (0.5 M NaCl, 1 M imidazole, 20 mM Tris HCl, pH 8.0) to elute bound proteins. SDS-PAGE is used for protein analysis.

### F-actin to G-actin ratio determination

The F-actin to G-actin ratio was determined by western blotting, as previously described (51). Briefly, the two forms of actin differed in that F-actin was insoluble, whereas G-actin was soluble. Coelomocytes were collected from the control, siAjNLR-new and siAjFidgetin treatment groups after *V*. *splendidus* infection. Equal numbers of coelomocytes were harvested in cold lysis buffer (10 mM K_2_HPO_4_, 100 mM NaF, 50 mM KCl, 2 mM MgCl_2_, 1 mM EGTA, 0.2 mM DTT, 0.5% Triton X-100, 1 mM sucrose, pH 7.0) and centrifuged at 15,000 g for 30 min. The amount of soluble actin (G-actin) in the supernatant was measured. The insoluble F-actin in the pellet was resuspended in lysis buffer plus an equal volume of buffer 2 (1.5 mM guanidine hydrochloride, 1 mM sodium acetate, 1 mM CaCl_2_, 1 mM ATP, 20 mM TrisHCl, pH 7.5) and incubated on ice for 1 h with gentle mixing every 15 min to convert insoluble F-actin into soluble G-actin. The samples were centrifuged at 15,000 g for 30 min, and the amount of F-actin in this supernatant was measured. Samples from the supernatant (G-actin) and pellet (F-actin) fractions were proportionally loaded and analyzed by western blotting using an actin-specific antibody (#MAB1501, 1:10,000, Millipore).

### Quantitative real-time PCR analysis

The transcripts of AjNLR-new and its coordinator genes were analyzed *via* quantitative real-time PCR (qRT-PCR) on the Applied Biosystem 7500 real-time PCR system. According to the manufacturer’s protocol, total RNA was extracted with the TRIzol reagent (Takara), and cDNA was prepared using PrimeScript RT reagent with gDNA Eraser Kit (Takara). Amplification was conducted in a 20 uL reaction volume containing 8 uL of 1:50 diluted cDNA, 0.8 uL of each primer (listed in Table S1), 10 uL of SYBR Green, and 0.4 uL of ROX (Takara). The reaction mixtures were incubated for 2 min at 95 °C, followed by 40 cycles of 15 s at 95 °C, 15 s at 60 °C, and 20 s at 72 °C followed by a melting curve. The baseline was automatically set by the software to maintain consistency. The relative expression levels were calculated using the 2^−ΔΔCT^ method with β-actin for normalization (52), each PCR trial was run in triplicate parallel reactions and repeated three times. The primer efficiency was checked. A significant difference in expression relative to expression in the control group at each time point is indicated using an asterisk for ∗*p* < 0.05 and two asterisks for ∗∗*p* < 0.01.

### Western blotting

The protein concentrations of coelomocyte lysates were determined with a BCA Protein Assay Kit (Sangon). Approximately 50 μg of protein was separated with 10% SDS-polyacrylamide gels and transferred to 0.45 μm ECL membranes. After blocking with 5% skim milk in TBST (50 mmol/L Tris-HCl, 150 mmol/L NaCl, and 1% Tween-20) at room temperature for 120 min, the membranes were incubated with antibodies diluted at 1:500 in 5% BSA solution at 4 °C overnight. The membranes were incubated with HRP-labelled anti-rabbit or mouse IgG (1:2000) in 5% BSA solution at room temperature for 1.5 h. The membranes were incubated in Western Lightning-ECL substrate (PerkinElmer) prior to exposure with X-OMAT AR X-ray film (Eastman Kodak). The densities of the protein bands were quantified using the ImageJcngr software package, and the results were derived from the statistical analysis of three independent experiments.

## Data availability

Requests for access to the data, statistical code, questionnaires, and technical processes may be made by contacting the corresponding author at lichenghua@nbu.edu.cn.

## Supporting information

This article contains supporting information.

## Conflict of interest

The authors declare that they do not have any conflicts of interest with the content of this article.
