# Supplementary material for: A unique echinoderm NLR triggers Vibrio phagocytosis by promoting microtubule severing to facilitate microfilament polymerization
Source: J Biol Chem. 2026 Apr 1;302(5):111418. doi: 10.1016/j.jbc.2026.111418 (PMC13133943; doi:10.1016/j.jbc.2026.111418)
Supplement: Supplementary information 1 [file mmc1.docx]

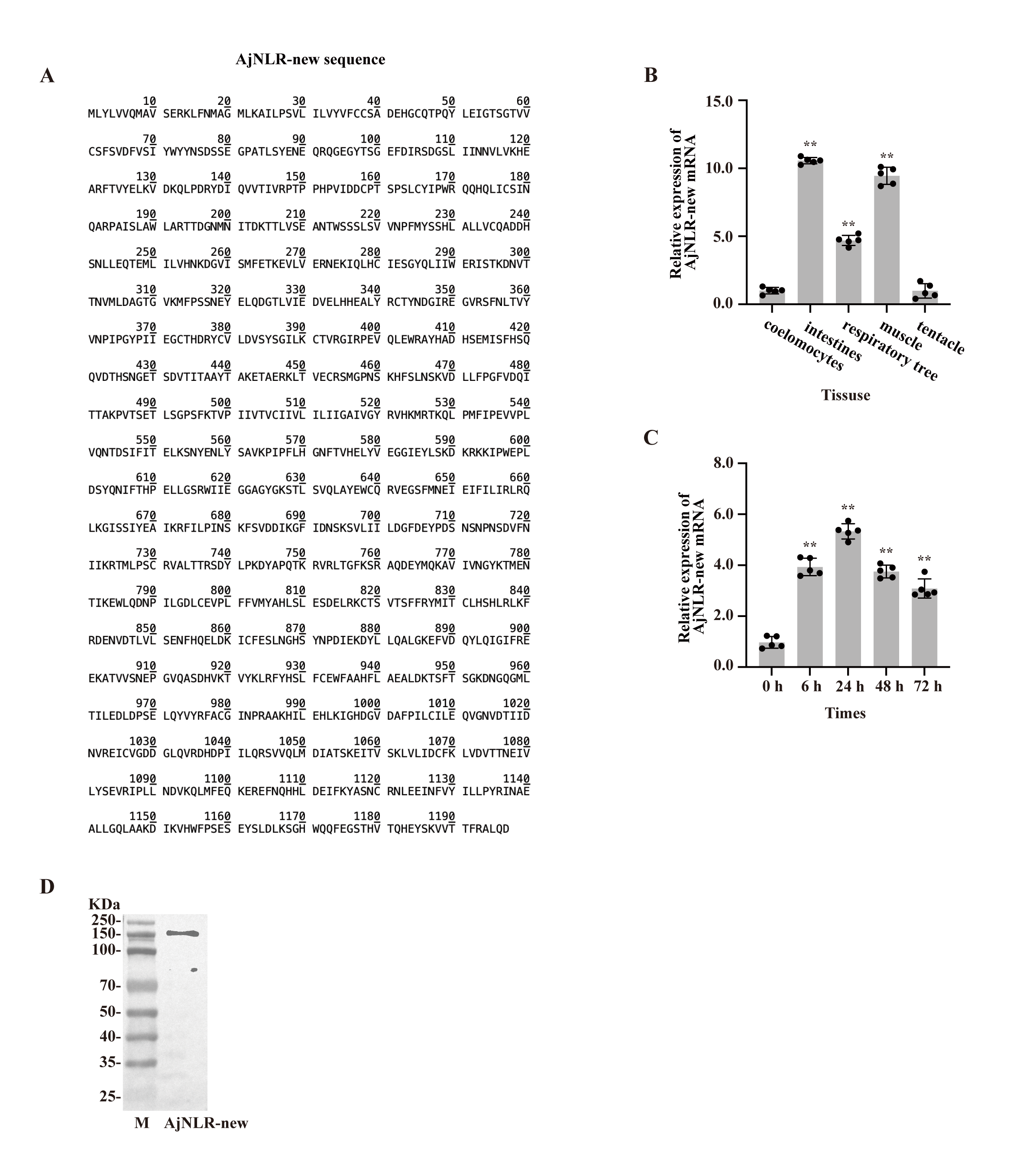


**Figure S1. Sequence characters of AjNLR-new.** (A) Amino acid sequences of the AjNLR-new. (B-C) The tissue distribution of AjNLR-new in healthy *A. japonicus* detected by qPCR. The transcript levels in intestine, muscle, tentacle, and respiratory tress were normalized to that in coelomocytes. Expression levels of AjNLR-new in *A. japonicus* coelomocytes infected with AJ01. mRNA expression level of AjNLR-new detected by qPCR. Student’s t-test (B, C) were used for statistical analysis. Data are expressed as mean ± SD of 5 independent experiments (B, C), ***p* < 0.01. (D) The specificity of AjNLR-new Ab verified by Western blotting with coelomocytes proteins.

**
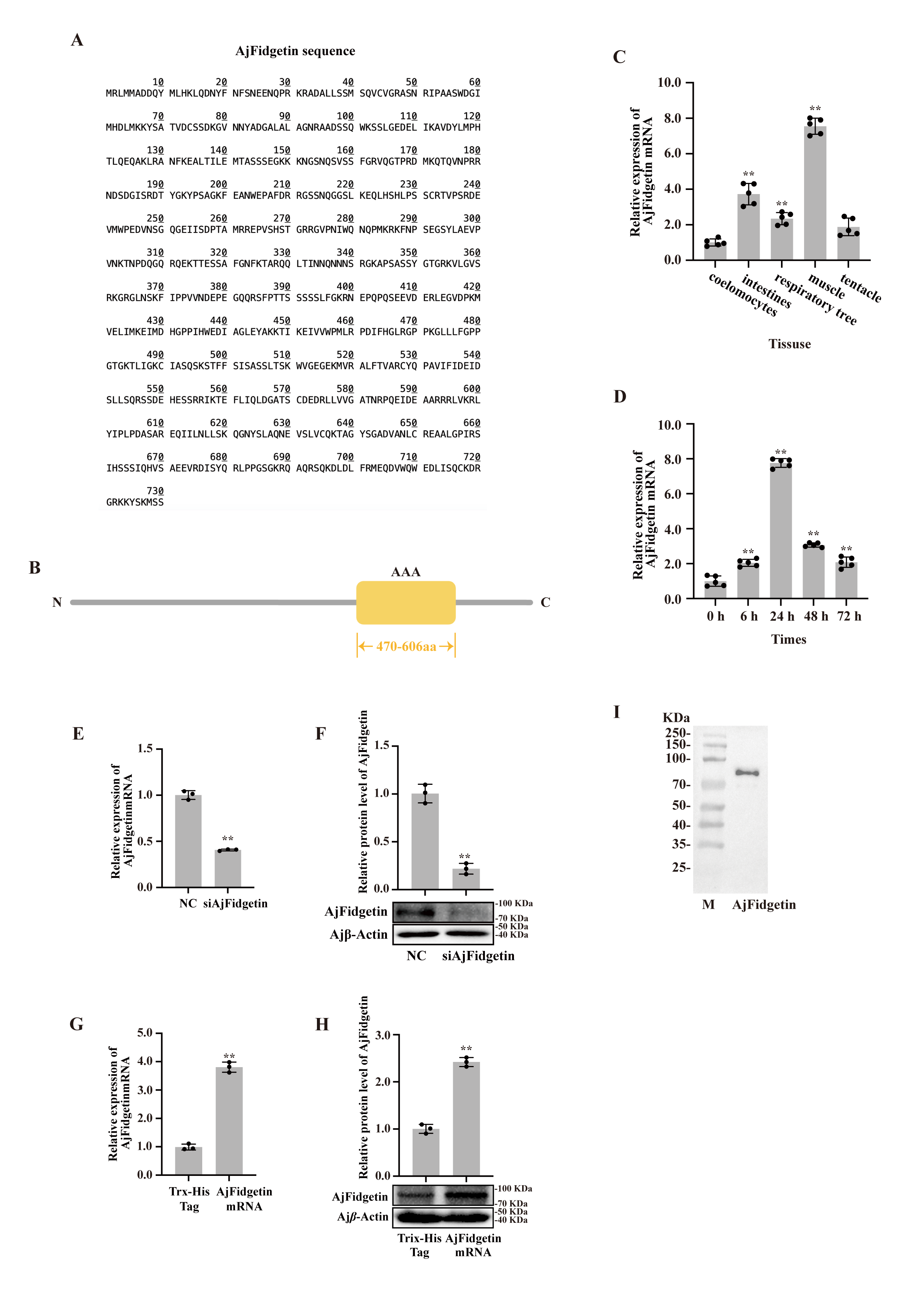
**

**Figure S2. Sequence characters of AjFidgetin.** (A) Amino acid sequences of the AjFidgetin. (B) The domain architecture of sea cucumber AjFidgetin predicted by SMART (http://www.smart.embl-heidelberg.de/). (C-D) The tissue distribution of AjFidgetin in healthy *A. japonicus* detected by qPCR. The transcript levels in intestine, muscle, tentacle, and respiratory tress were normalized to that in coelomocytes. Expression levels of AjFidgetin in *A. japonicus* coelomocytes infected with AJ01. mRNA expression level of AjFidgetin detected by qPCR. (E-F) The efficiency of siAjFidgetin in coelomocytes was determined using qPCR and western blotting analysis. (G-H) The efficiency of overexpression AjFidgetin in coelomocytes was determined using qPCR and western blotting analysis. The protein expression pattern was digitalized using Image J software by scanning the western blotting bands. The relative expression levels of AjFidgetin /β-Actin were expressed as the mean ± SD, and the value of the NC or Trx-His Tag was set as one. Student’s t-test (C, D, E, G) were used for statistical analysis. Data are expressed as mean ± SD of 5 independent experiments (C, D, E, G), ***p* < 0.01. (I) The specificity of AjFidgetin Ab verified by Western blotting with coelomocytes proteins.

**Table S1. Primers used in this study**

| Primers | Sequences(5’-3’) | Used for |
| --- | --- | --- |
| *Aj*NLR-new F | ATGTTGTACCTTGTAGTACAAAT | Recombinant expression |
| *Aj*NLR-new R | TTAGTCCTGCAATGCACGGAAGGT |  |
| *Aj*NLR-new-EX F | GGTAGTAGATGGATCATTGAAGGAGGG |  |
| *Aj*NLR-new-EX R | TACGATCACGGCTTTTTGCATGTAT |  |
| *Aj*NLR-new-IN F | GAACATGGTTGTCAAACTCCGCAGT |  |
| *Aj*NLR-new-IN R | CCCAGGGATTGGATTCACATATACA |  |
| *Aj*Fidgetin F | ATGCGTTTGATGATGGCTGATGAT |  |
| *Aj*Fidgetin R | CTATGAAGACATCTTTGAATATTTCTTCCT |  |
| *Aj*Fidgetin-AAA F | CCGATGCTTAGACCAGACATTTTTCA |  |
| *Aj*Fidgetin-AAA R | GTTGAGGATGATTTGCTCTCGAGCCGA |  |
| *Aj*β-Tubulin F | ATGCGTGAAATCGTCCATATTCAAGCA |  |
| *Aj*β-Tubulin R | TTAAGCAGCCTCATCTTCTTCCTCCTCC |  |
| *Aj*NLR-new qF | TAGAAGATGTGGAACTACACCACGA | Real-time PCR |
| *Aj*NLR-new qR | CTGCGTGGTATGCTCTCCATTCTAA |  |
| *Aj*Fidgetin qF | CAGAACCAGCCGATGAAGAGAAAAT |  |
| *Aj*Fidgetin qR | GGTGCCTTTCCTCTTGAGTTGTTGT |  |
| *Aj*β-actin qF | CCATTCAACCCTAAAGCCAACA |  |
| *Aj*β-actin qR | ACACACCGTCTCCTGAGTCCAT |  |
| si*Aj*NLR-new-1 | CCGGCUAACAGGAUUUAAATT | RNA interference |
| si*Aj*NLR-new-2 | CUCCCUGUCUGUUGUUAAUTT |  |
| si*Aj*Fidgetin-1 | CGAGGUGCCAGUUAAUAAATT |  |
| si*Aj*Fidgetin-2 | GCCUCGCAAUCCAAAUCAATT |  |
| siRNA-NC  (Negative control) | UUCUCCGAACGUGUCACGUTT |  |
|  | ACGUGACACGUUCGGAGAATT |  |
